# Effects of Stress on Alcohol Consumption

**DOI:** 10.35946/arcr.v34.4.01

**Published:** 2012

**Authors:** Robert Anthenelli, Lindsey Grandison

**Affiliations:** **Robert M. Anthenelli, M.D.,***is associate chief of staff for Mental Health, Veterans Affairs, San Diego Healthcare System; professor and vice chair for Veterans Affairs; and director, Pacific Treatment and Research Center, Department of Psychiatry, School of Medicine, University of California, San Diego, California.*; **Lindsey Grandison, Ph.D.,***is a program director in the Division of Neuroscience and Behavior, National Institute on Alcohol Abuse and Alcoholism, Bethesda, Maryland.*

This issue of *Alcohol Research: Current Reviews* focuses on the impact of stress on alcohol consumption. The significance of stress on alcohol abuse recently has been reemphasized by the alcohol use problems following post-traumatic stress disorder, such as those seen with some combat veterans. Behavior is described as an interaction between genetic constitution and environmental influences. Of the environmental factors affecting an individual, one of the most potent is external stress. Although it generally is held that stress increases drinking, the articles in this issue clearly demonstrate the complexities of this simple construct. It now is appreciated that the notion of stress itself is multidimensional. Early-life stressors such as child abuse can cause delayed and long-term consequences. The stress resulting from a traumatic event, either personal or public, such as an earthquake, can produce changes in drinking behavior. The effect of cumulative stressors throughout life can impact drinking as well. In addition to the dynamics of when stress is experienced, the type of stressor and the genetic constitution of the individual, as well as the stage of alcohol exposure can influence the response to stress. To a social drinker stress can have a different impact than stress for an abstinent alcoholic struggling with relapse. These factors now are better appreciated in the interaction between stress and alcohol use disorders and may help to decipher the often conflicting and contradicting observations found in the literature on this subject.

The connection between stress and alcohol consumption was made early on in alcohol research (Horton 1943). In the tension-reduction hypothesis, stress was seen to increase anxiety, and in response alcohol was consumed to reduce the anxiety. This connection between stress and alcohol was further linked by observations showing that in alcoholics the physiological responses to stress were perturbed. These stress actions involved the hypothalamic–pituitary–adrenal axis. Chronic alcohol consumption is associated with elevated basal glucocorticoid secretion, whereas the hormonal response to a stressor was blunted. In addition, a high dose of alcohol increases the adrenal hormone glucocorticoid. Following these observations, a body of evidence was generated in rodents to suggest that the increase in glucocorticoid would increase drinking. Subsequent findings have implicated other/additional circuits for connecting stress with alcohol use. Stress response also is mediated by the amygdala. Chronic alcohol exposure alters amygdala function, leading to increased corticotropin-releasing factor expression in the amygdala. This neuroadaptation is proposed to produce an altered affective state. Alcohol initially is able to ameliorate this effect and thereby provides a motivation for continued alcohol consumption. Furthermore, stress also affects the prefrontal cortex, reducing its capacity for executive function and resulting in augmented impulsivity.

At present epidemiological data support a link between stress and alcohol use disorders. However, the connection is not predictably causal. Stress under all circumstances does not necessarily lead to alcohol consumption. Genetic factors and past history of life experiences can influence this interaction. These complexities are abundantly exemplified in the experimental animal literature. Perhaps the greatest limitation in investigating the link between stress and alcohol use is the absence of a simple animal model in which a stressor results in a substantial increase in consumption over a sustained period of time. Of similar difficulty is establishing models of stress with full relevance to alcoholics. Financial issues, job loss, divorce, and other events are the day-to-day relevant stressors for human populations in developed countries. How these experiences are modeled in animal studies that are necessary for examining the neurobiological mechanisms involved currently is unresolved.

Future studies taking advantage of better genetic models, neuroimaging in human and animal studies, and findings on epigenetic modifications promise to clarify the linkage between stress and alcohol abuse disorders and help to show where and when stress will affect drinking behavior. Such information should provide targets for effective medication development.
